# Assessment of Fractional Factorial Design for the Selection and Screening of Appropriate Components of a Self-nanoemulsifying Drug Delivery System Formulation

**DOI:** 10.15171/apb.2019.070

**Published:** 2019-10-24

**Authors:** Ilham Kuncahyo, Syaiful Choiri, Achmad Fudholi, Ronny Martien, Abdul Rohman

**Affiliations:** ^1^Faculty of Pharmacy, Gadjah Mada University, Sekip Utara, Yogyakarta, Indonesia 55281.; ^2^Department of Pharmaceutical Science, Setia Budi University, Mojosongo, Surakarta, Indonesia 57127.; ^3^Pharmaceutical Technology and Drug Delivery, Department of Pharmacy, Universitas Sebelas Maret, Surakarta, Indonesia 57126.; ^4^Department of Pharmaceutics, Gadjah Mada University, Sekip Utara, Yogyakarta, Indonesia 55281.; ^5^Department of Pharmaceutical Chemistry, Gadjah Mada University, Sekip Utara, Yogyakarta, Indonesia 55281.

**Keywords:** Fractional factorial design, SNEDDS, Screening, Optimization, Statistical approach

## Abstract

***Purpose:*** Recently, a self-nanoemulsifying drug delivery system (SNEDDS) has shown great improvement in the enhancement of drug bioavailability. The selection of appropriate compositions in the SNEDDS formulation is the fundamental step towards developing a successful formulation. This study sought to evaluate the effectiveness of fractional factorial design (FFD) in the selection and screening of a SNEDDS composition. Furthermore, the most efficient FFD approach would be applied to the selection of SNEDDS components.

***Methods:*** The types of oil, surfactant, co-surfactant, and their concentrations were selected as factors. 2^6^ full factorial design (FD) (64 runs), 2^6-1^ FFD (32 runs), 2^6-2^ FFD (16 runs), and 2^6-3^ FFD (8 runs) were compared to the main effect contributions of each design. Ca-pitavastatin (Ca-PVT) was used as a drug model. Screening parameters, such as transmittance, emulsification time, and drug load, were selected as responses followed by particle size along with zeta potential for optimized formulation.

***Results:*** The results indicated that the patterns of 2^6^ full FD and 2^6-1^ for both main effects and interactions were similar. 2^6-3^ FFD lacked adequate precision when used for screening owing to the limitation of design points. In addition, capryol, Tween 80, and transcutol P were selected to be developed in a SNEDDS formulation with a particle size of 69.7± 5.3 nm along with a zeta potential of 33.4± 2.1 mV.

***Conclusion:*** Herein, 2^6-2^ FFD was chosen as the most efficient and adequate design for the selection and screening of SNEDDS composition. The optimized formulation fulfilled the requirement of a quality target profile of a nanoemulsion.

## Introduction

Recently, bioavailability enhancement of poorly water soluble or poorly absorbed drugs has become an interesting and promising area in pharmaceutical research.^1,2^ Although many approaches have been introduced, several of them have failed during the scale-up process owing to issues related to cost effectiveness or feasibility.^3,4^ Lipid formulations have several advantages, including low cost, high drug load, high efficiency, and feasibility in terms of scaling up.^1,5^ Therefore, lipid formulation-based nanoemulsions using self-emulsifying mechanisms are increasingly being developed because of their flexibility. A self-nanoemulsifying drug delivery system (SNEDDS) could be used to deliver either lipophilic or hydrophilic drugs.^6,7^ SNEDDS can be modified to enhance the permeability of hydrophilic drugs.^8^ Specifically, SNEDDS are preconcentrates of nanoemulsion comprising organic components, including oil, surfactants, or additional co-surfactants/co-solubilizers in the mixture. When a preconcentrate is diluted with water under gentle agitation, it forms ultrafine dispersion of oil in water through a self-driven mechanism.^9-11^

The successful formulation of SNEDDS and its self-emulsifying mechanism are dependent on its components, namely oil, surfactants, and co-surfactants.^12,13^ Oil droplets are a carrier dispersed ultrafinely, and their interfacial tension is stabilized by surfactants and co-surfactants.^14^ Surfactants are the main components that determine the self-emulsification mechanism and droplet size.^15-18^ A co-surfactant works as a solubilizing agent of the drug in oil and possibly helps the surfactant to stabilize the oil dispersion.^12,19^ Failure in the selection of appropriate oil, surfactants, and co-surfactants leads to errors in SNEDDS formulations. Several studies have reported that the screening of SNEDDS composition was dependent on the solubility of the drug in each component.^13,20^ The selection of each oil, surfactant, and co-surfactant could be optimized using a pseudo-ternary component diagram.^6,13,21,22^ However, this method has the following disadvantages. The material selected based on drug solubility does not directly correlate with the self-emulsification mechanism, i.e., affinity between oil and surfactant/co-surfactant, and required one or more steps, i.e., determination of composition or range of concentration, to produce a nanodispersion by a self-driven mechanism, depending on the ternary component diagram.^19^

Nanoemulsion with SNEDDS not only consists of oil, surfactants, and co-surfactants, but also water as a medium to dilute and produce nanodroplet dispersions.^15^ Thus, there are four components needed to construct a ternary diagram, and consequently several runs must be performed.^23-25^ In addition, the miscibility of components is a major factor involved in achieving a successful formulation, and it is not correlated with drug solubility in each component.^24,25^ Furthermore, in this study, a simultaneous approach using statistical-based screening methods was introduced during the screening of SNEDDS components.

Fractional factorial design (FFD), based on the full factorial design (FD) but with a reduction in terms of design point under fraction levels, can be used to select and assess certain factors contributing to successful SNEDDS formulations.^26^ To the best our knowledge, no study has reported on the application of FFD for screening and selecting appropriate components during SNEDDS formulation. FFD has been successfully applied for choosing the appropriate conditions for Cu(II) extraction from soybean-based organic solvent,^27^ robustness testing of analytical methods,^28,29^ assessing the effect of process parameters,^30,31^ and determining optimized conditions for material synthesis.^32^

In this study, we used pitavastatin (PVT) as a drug model, and it was incorporated into a lipid-SNEDDS formulation. Ca-pitavastatin (Ca-PVT), a stable form of PVT, has very low solubility in water, a molecular weight of more than 500 Da, a polar surface area greater than 140 Å^2^, and a rotatable bound of more than 12. According to the Lipinski and Veber rules, oral absorption of Ca-PVT is onerous.^33^ Therefore, PVT is a suitable and promising candidate to be formulated in a SNEDDS context. Hence, this study compared the adequacy of each level of FFD using six factors under 2-level (2^6-1^, 2^6-2^, and 2^6-3^ FFD) to 2^6^ full FD. The most efficient and appropriate FFD approach would be applied to the selection of SNEDDS components.

## Material and Methods

### Materials

Capryol-90 and Transcutol P were obtained from Gattefose (Saint Priest, France). Oleic acid and Tween 80 were purchased from Sigma Aldrich (St. Louis, USA) and Kolliphor EL was procured from BASF (Ludwigshafen, Germany). Ca-PVT was purchased from Thanen Chemical Co. Ltd. (Xinbei Distrcit, China).

### Experimental design for screening of SNEDDS components

Various schemes of FD were implemented in this study, namely 2^6^ full FD, 2^6-1^ FFD, 2^6-2^ FFD, and 2^6-3^ FFD. Numerous factors, including numerical and categorical factors, were involved in this study - types of organic compositions (oil, surfactants, and co-surfactants) and ratio of each organic component. Each factor has two levels, just as presented in Table 1. Each model was evaluated statistically and compared with one another employing multiple linear regression analysis (MLRA) and fitted using the following equation:

**Table 1:** Designed factors and levels of 2^6^ full factorial design and fractional factorial design

**Level**	**Factors**					
	**Categorical**			**Numeric** ^a^		
	**A**	**B**	**C**	**D**	**E**	**F**
Low level (-1)	Capryol	Kolliphor EL	PEG 400	1	3	1
High level (+1)	Oleic acid	Tween 80	Transcutol CG	3	6	4

^a^ Calculated based on weight of each component to total weight ratio (total weight = 10 g). A = oil, B = surfactant, C = co-surfactant, D = oil weight ratio, E = surfactant weight ratio, F = co-surfactant weight ratio.

Y = a + m_1_*A + m_2_*B + m_3_*C +m_4_*D + m_5_*E + m_6_*F + i_1,2_*A*B + i_1,3_*A*C + i_1,4_*A*D + i_1,5_*A*E + i_1,6_*A*F + i_2,3_*B*C+ i_2,4_*B*D + i_2,5_*B*E + i_2,6_*B*F+ i_3,4_*C*D + i_3,5_*C*E + i_3,6_*C*F + i_4,5_*D*E + i_4,6_*D*F + i_5,6_*E*F(1)

where *a* is the intercept; *A*, *B*, *C*, …, and *F* are coded levels of each level; *m*_1_, *m*_2_, *m*_3_, …, and *m*_6_ are regression coefficients of the main effect; and *i*_1,2_, *i*_1,3_, *i*_1,4_, …, *i*_5,6_ are regression coefficients of the interaction of the two factors.

A 2^6^ full FD consisting of 64 runs was utilized in this study. The design point of FFD was obtained from the result of the 2^6^ full FD. The design points of a 2^6^ full FD, 2^6-1^ FFD, 2^6-2^ FFD, and 2^6-3^ FFD are presented in Table S1 (see Supplementary File 1).

### Saturated SNEDDS preparation

Oil, surfactants, and co-surfactants were weighed separately (total 10 g) depending on their composition (presented in Supplementary file 1) and mixed together using an ultrasonicator and stirrer. An excess amount of PVT (1-2 g) was added until a saturated condition was achieved (mixing for approximately 72 h at ambient temperature, 26 ± 1°C). The mixture was centrifuged at 12 000 *×*g for 30 minutes and the supernatant was collected as a saturated SNEDDS formulation and stored until use for further characterization.

### Characterization of saturated SNEDDS formulation

A saturated SNEDDS formulation was characterized using transmittance (%T), emulsification time (ET), and drug load (DL) as screening parameters. The parameters chosen could be used to depict the quality of target profiles of the SNEDDS formulation.

ET was measured by diluting SNEDDS 100 times. Briefly, one part of the SNEDDS was added to 100 parts of water at 37 ± 2°C and an agitation speed of 100 rpm. The time required to obtain ultrafine and homogenous droplet dispersion in water was noted as ET. Then, the dispersion was stirred at 500 rpm for 5 min. %T was scanned using a Hitachi U-2900 spectrophotometer (Kyoto, Japan) at 650 nm. Each design point was carried out in triplicate.

DL was determined by the amount of drug (mg) that could be loaded in 1 g of a lipid formulation mixture. A sample was taken, diluted with methanol, and scanned spectrophotometrically at 244 nm through a validated analytical method (linearity, accuracy, precision, and placebo interference). Once more, each design point was carried out in triplicate.

### Statistical analysis

The obtained data were summarized and categorized according to each fraction of FFD (Supplementary file 1 , Table S2). The data of each design was fitted to Eq. 1. The models, including the intercept, main effect, and interactions, were generated for all response variables via an MLRA approach. The models were evaluated based on several statistical parameters, including coefficient of determination (R^2^), adjusted coefficient of determination (Adj. R^2^), predicted coefficient of determination (Pred. R^2^), adequate precision (adeq. prec), and predicted residual error sum of squares (PRESS). All parameters were analysed with Design Expert^®^, version 10 (Stat-Ease Inc., Minneapolis, MN), software. Depending on their main effects (*m*) and interactions, the contribution of each main effect was calculated according to the following equation:

(2) $$Main\;effect\;contribution(\%)=\frac{m_{x}}{\sum_{6}^{1}m}\times 100\%$$

The main effect contributions involving a two-factor interaction model was calculated based on the percentage of regression coefficient divided by the sum of regression coefficients. All main effect contributions of FFD were compared to the 2^6^ full FD statistically. Principal component analysis (PCA) was applied to generate the score plot of each design. The distance between the fractional designs and 2^6^ full FD was measured using the root mean square of residual (RMSR) values calculated via the equation:

(3) $$RMSR=\sqrt{\frac{\sum_{6}^{1}\left|x-x_{i}\right|}{N-2}}$$

where *x* is the contribution effect of the 2^6^ full FD and *x*_i_ is the contribution effect of fractional designs. Residual threshold lines of 20 and 30% were constructed to obtain the alert limit of error. A contour plot was generated from the MLRA and compared to each design model.

### Optimization and characterization

An optimized formulation was derived with the 2^6^ factorial design. %T, ET, and DL were selected to be combined as an overlay plot. The optimized formulation purposefully had a particle size < 100 nm and a physically stable formulation (zeta potential at less than -30 mV). Therefore, the optimized formulation was characterized using a dynamic light-scattering technique for particle size along with an electrophoretic mobility for zeta potential measurement employing a Horiba SZ-100 particle size analyser (Kyoto, Japan). The optimized formulation was diluted 100 times with water and introduced into a glass cuvette. The sample was measured at a wavelength of 632 nm, angle of 173°, and a refractive index of 1.333, while sample absorbance was adjusted according to the absorbance of the sample. Gate time was adjusted within a range of 2.56 to 10.24 µs to achieve appropriate conditions for measurement. Zeta potential was assessed with a carbon cuvette at 25°C.

## Results and Discussion

In a prior study, we performed the selection of appropriate components for the SNEDDS formulation depending on drug solubility. However, there was no efficiency determined if each selected component that had the highest solubility was not miscible or did not form an isotropic mixture. Thus, many runs would be performed to yield the selected material, which had miscibility and could form nano-sized regions when it was diluted with water.

FFD appears to be the most efficient method for screening and selecting the appropriate components for SNEDDS formulations. FFD has an advantage over other techniques in that not only numerical factors, but also categorical factors,^30,34^ such as the types of oil, surfactant, and co-surfactant, could be tested. The 2^6^ full FD consisted of 64 runs, and the first fraction, 2^6-1^ FFD, reduced to half of the full design (32 runs). In addition, the second fraction, 2^6-2^ FFD, reduced to a quarter of the full design (16 runs) while the last fraction, 2^6-3^ FFD, diminished to one eighth of the full design (eight runs) (Table S1). The reduction in factors had a positive impact that enhanced efficiency through reduction in cost and time owing to the decrease in number of runs.^26^ Missing information or irregularity in information that correlated with either effects or interactions could be obtained,^31^ so, therefore, the evaluation was carried out based on the main effect contribution of each factor.

MLRA was applied to generate the equation. The goodness-of-fit parameters, namely R^2^ more than 0.7, the difference between Adj. R^2^ and Pred. R^2^ less than 0.2, and adequate precision of more than 4 were used as criteria for the selection of an appropriate model.^35^ All models were significant (*P* < 0.05) and a lack-of-fit test indicated non-significance (*P* > 0.05). SNEDDS composition affected the self-emulsifying process owing to the contribution of hydrophilicity or hydrophobicity and the relevant characteristics. Thus, ultrafine droplet dispersion was the main objective in the selection of SNEDDS composition.^17,19^ The colour of the nanoemulsion depicts the formation of a miscellar solution, nano/micro emulsion, or macro emulsion. When diluted with a medium (e.g., water or simulated gastro-fluid/intestinal; SGF/SIF), the miscellar solution usually had a clear appearance, while the nano/micro emulsion had a bluish or translucent appearance and the macro emulsion exhibited turbidity, followed by separation of the oil phase based on poor stability.^36^ Therefore, in this study, %T was proposed as a parameter to characterize the formation of droplet dispersion because it is cheap, fast, and feasible during the screening process.^37^ In addition, there was no assessment of size distribution with respect to its impact on SNEDDS because of the enhancement of efficiency during the screening process.

**Figure 1:**
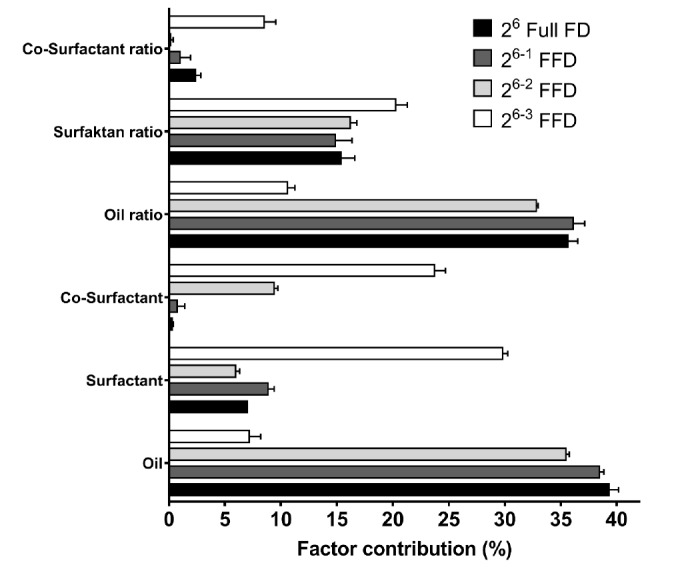
Contribution of each factor of 2^6^ full factorial design (FD), 2^6-1^ fractional factorial design (FFD), 2^6-2^ FFD, and 2^6-3^ FFDon the transmittance.

The observed values for all calculations are presented in Table S2. Based on MLRA of the %T value of each design, the contribution of each factor is found in Figure 1. Therein is depicted the percentage of factor contribution to %T. In addition, the pareto chart of %T is portrayed in Figure S1. Both oil type and oil ratio had the greatest effect on %T. The molecular structure or type of oil in the nanoemulsion is a crucial component that has gained attention from researchers seeking to determine globule size.^15^ Meanwhile, the co-surfactant had a negligible effect on %T. The reduction in runs, or in other words, an increase in the number of fractionated factors (2^6-1^–2^6-3^ FFD) altered the effects of such a contribution. Almost similar patterns were observed for 2^6^ full FD, 2^6-1^, and 2^6-2^ FFD. Depending on the regression coefficient of MLRA, we could explain the effect on and interaction between factors (Table 2). With this, oil had a significant effect on %T. The oil phase in a nanodroplet is the core of the carrier. The type and concentration of this component in the system plays a fundamental role in determining droplet size.^12,19^ In this study, we compared hydroxylated oil (non-water-soluble) and pure fatty acid. The results showed that better and finer droplets were achieved without producing a macro droplet or separating its phase. The hydroxylated oil functioned as a surfactant owing to the presence of hydrophilic groups. Despite the contribution of hydrophilic groups, hydroxylated oil had little impact on water solubility. Thus, hydroxylated oil helped in the formation of droplets depending on its ability to regulate interactions with the surfactant, specifically when the lipid formulation was diluted with a medium. The surfactant ratio in the SNEDDS composition has a marked effect on the stabilization of oil droplet dispersion. Moreover, the surfactant ratio had greater effects than surfactant type.^15,36,38^

**Table 2:** Statistical parameters of transmittance (%T), emulsification time (ET), and drug load (DL) using 2^6^ full factorial design (FD), 2^6-1^ fractional FD (FFD), and 2^6-2^ FFD

**Regression code**	**2** ^ 6 ^ **Full FD**			**2** ^ 6-1^ **FFD**			**2** ^ 6-2^ **FFD**		
	**%T**	**ET**^-1/2^**x10**^-2^	**log(DL) x10**^-2^	**%T**	**ET**^-1^**x10**^-3^	**DL**	**%T**	**Log(ET)**	**DL**^-1^**x10**^-3^
Intercept	54.70	21.00	195.00	54.09	56.0	96.40	53.35	1.46	13.00
A	-20.71	-4.90	-1.20*	-19.90	-21.0	1.31*	-19.81	0.29	-0.07*
B	-3.66	0.33*	4.00	-4.58	1.3*	10.34	-3.34	-0.06	-0.17*
C	0.06*	3.30	12.00	0.37*	16.0	21.89	-5.24	-0.14	-2.14
D	-18.77	-2.30	6.30	-18.69	-10.0	12.62	-18.34	0.09	-0.41*
E	8.10	0.69*	-5.70	7.70	4.6	-12.49	9.06	-0.02*	0.29*
F	1.27*	3.60	2.10	0.34*	16.0	7.86	-0.07*	-0.2	-0.61*
AB	3.67	0.57*	1.60*	3.54	-1.9*	-2.11*	-	-	-
AC	-0.37*	-1.50	-0.99*	0.056*	-13.0	-1.47*	-	-	-
AD	0.42*	1.80	3.50	1.42	5.8	3.81*	-	-	-
AE	1.17*	0.16*	0.65*	0.76*	-1.4*	-4.58	-	-	-
AF	0.22*	-0.59*	-0.66*	1.97	-5.89	-10.45	-	-	-
BC	-1.79	0.33*	5.40	-1.21*	1.1*	13.28	-	-	-
BD	-4.00	-1.70	-0.06*	-3.69	-4.8	5.33	-	-	-
BE	1.94	1.20	2.90	1.66*	-0.5*	5.87	-	-	-
BF	0.23*	0.19*	2.90	-0.12*	-0.4*	6.84	-	-	-
CD	-0.48*	-1.40	-1.50*	0.26*	-9.6	-3.16*	-	-	-
CE	0.48*	-0.19*	-0.86*	1.24*	0.4*	-5.17	-	-	-
CF	-0.49*	2.30	5.40	-4.17	6.6	20.31	-	-	-
DE	1.51	-0.53*	-4.40	1.29*	-3.3*	-5.75	-	-	-
DF	0.84*	-1.20	-4.00	-0.68*	-7.4	-5.04	-	-	-
EF	0.073*	0.02*	2.90	0.90*	8.8	4.88	-	-	-
R^2^	0.9239	0.7779	0.7329	0.9442	0.8817	0.8765	0.9122	0.8441	0.344
Adj. R^2^	0.9145	0.7504	0.6999	0.9284	0.8481	0.8414	0.8993	0.8213	0.248
Pred. R^2^	0.903	0.7167	0.6592	0.9061	0.8008	0.7921	0.8796	0.7863	0.1009
Adeq. Prec	35.83	23.627	15.684	27.033	22.845	18.462	26.245	19.19	5.444

* Not significant difference (*P* < 0.05).

**Figure 2:**
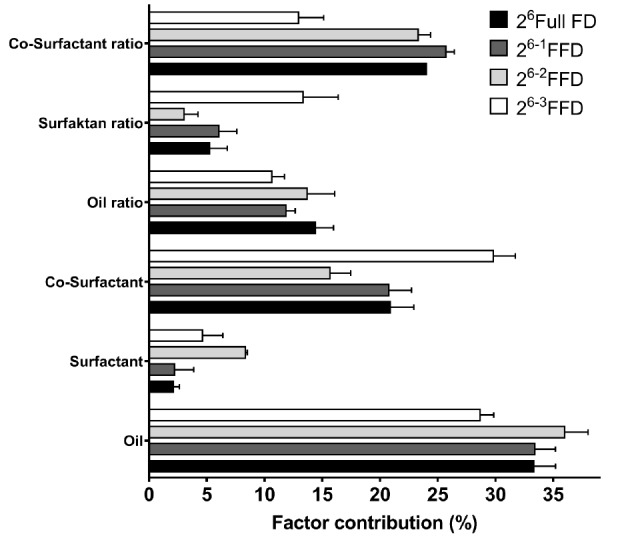
Contribution of each factor of 2^6^ full factorial design (FD), 2^6-1^ fractional factorial design (FFD), 2^6-2^ FFD, and 2^6-3^ FFDon the emulsification time.

The faster the dispersion of droplets when a lipid formulation is introduced to the medium, the higher the requirement of the quality target profile of the SNEDDS formulation. Therefore, the time required to emulsify the lipid formulation completely under gentle agitation was a main feature considered for the selection of appropriate components in SNEDDS formulations. In addition, the longer the ET, the lower the possibility of forming ultrafine droplet dispersions. ET was not only affected by hydrophilicity and hydrophobicity, but also by viscosity and the density of lipid formulation.^39^ The main effect contribution of ET is illustrated in Figure 2, and the MLRA of ET is presented in Table 2. In addition, the pareto chart of ET is illustrated in Figure S2.

The oil type made the most pronounced contribution to ET. However, different types of oils had varying effects on ET, as mentioned before. Pure fatty acid had higher lipophilicity than hydroxylated oil; therefore, it promoted longer ET. In this system, both surfactant type and ratio had negligible effects on ET. Meanwhile, the co-surfactant had the second highest impact on ET after oil type. The hydrophilic characteristics of co-surfactants modified the hydrophilicity of the lipid formulation. In addition, the presence of a hydrophilic co-surfactant caused the lipid formulation to disperse readily with water.^24^ In this study, we demonstrated that the selection of appropriate components in SNEDD formulations, especially for co-surfactants, not only aided the surfactant in stabilizing the oil dispersion, but also in modifying hydrophilicity.

**Figure 3:**
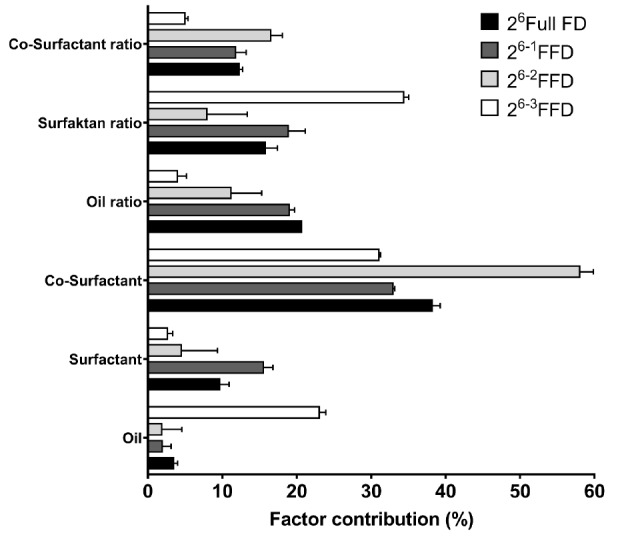
Contribution of each factor of 2^6^ full factorial design (FD), 2^6-1^ fractional factorial design (FFD), 2^6-2^ FFD, and 2^6-3^ FFDon the pitavastatin loading.

A drug is incorporated into a SNEDDS formulation either within an oil droplet or in the hydrophobic region of a surfactant. However, the ability to load the drug is the main capability of a delivery system in achieving the therapeutic objectives owing to correlating with potency and acceptability.^25^ The MLRA of DL is located in Table 2. The contribution effect of DL was generated and is shown in Figure 3. In addition, the pareto chart of DL is presented in Figure S3. Co-surfactant demonstrated the greatest contribution toward increasing DL. Different co-surfactant types had a distinct effect on DL based on solubility power.^24,36^ In this study, transcutol and monoethyl diglycol, had higher solubility power than polyethylene glycol. Nevertheless, oil, surfactant, and co-surfactant ratios had a similar contribution effect to DL. Of note, an increase in surfactant ratio reduced DL.

**Figure 4:**
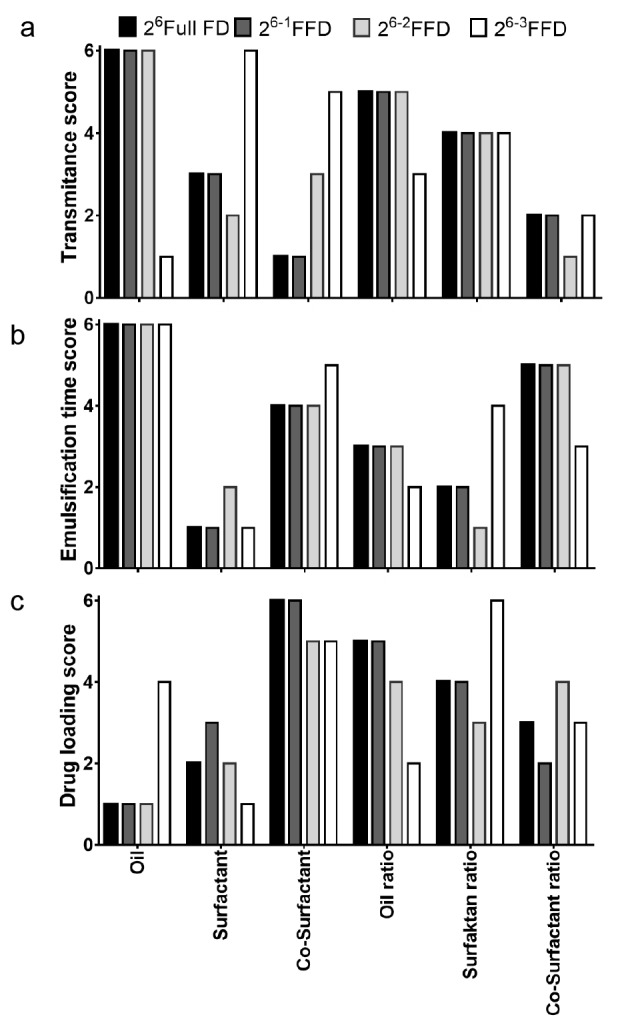
Score of factor contribution of transmittance (a), emulsification time (b), and drug loading (c).

**Figure 5:**
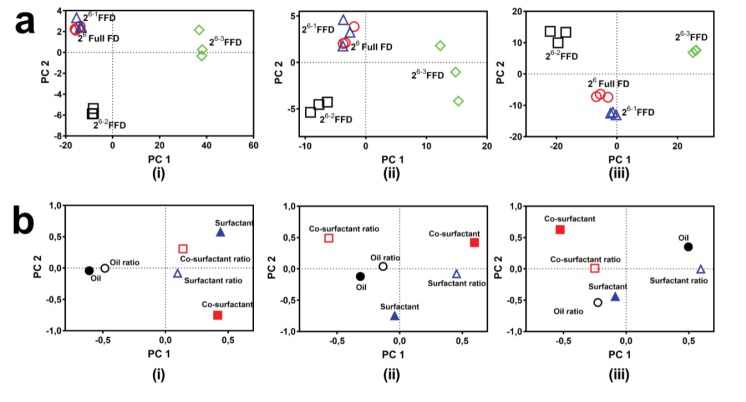
Principal component analysis: Score plot (a) and loading plot (b) of %transmittance (i), emulsification time (ii) and drug load (iii). 2^6^ full factorial design (FD) (O), 2^6-1^ fractional factorial design (FFD) (△), 2^6-2^ FFD (□), and 2^6-3^ FFD (⋄).

To select the appropriate composition of SNEDDS formulation qualitatively, we arranged, depending on the ranking of each contribution factor, %T, ET, and DL (Figure 4). Depending on the ranking pattern, similar patterns could be yielded for 2^6^ full FD, 2^6-1^ FFD, and 2^6-2^ FFD. However, with the DL score pattern, a slightly dissimilar pattern was obtained for the 2^6-2^ FFD. In addition, as the 2^6-3^ FFD altered the ranking pattern of the contribution effect, it could not be used because of a lack of adequate main effect prediction in the ranking pattern. Insignificant terms caused a bias effect in determining the rank of the contribution effect. Therefore, this negligible contribution effect was ignored. PCA is used to reduce the number of predictors in a multivariate analysis; therefore, it can be used to reach a conclusion depending on score value.^40^ The PCA score plot for %T, ET, and DL is presented in Figure 5a. Reduction of half design (2^6-1^ FFD) had a similar score in terms of PCA to full design (2^6^ full FD). This was demonstrated by the closeness-of-score plot points. 2^6-2^ FFD had a relatively long distance, but a longer distance from full design was observed for 2^6-3^ FFD. The loading plot (Figure 5b) exhibited a contribution to each factor when determining the difference of each design. Both oil type and ratio determined transmittance value. Meanwhile, co-surfactant ratio promoted a higher factor when determining emulsification time and drug loading. In addition, the surfactant ratio also had a major contribution to DL. All of these phenomena were similar to several reported studies.^8,9,19,21^

**Figure 6:**
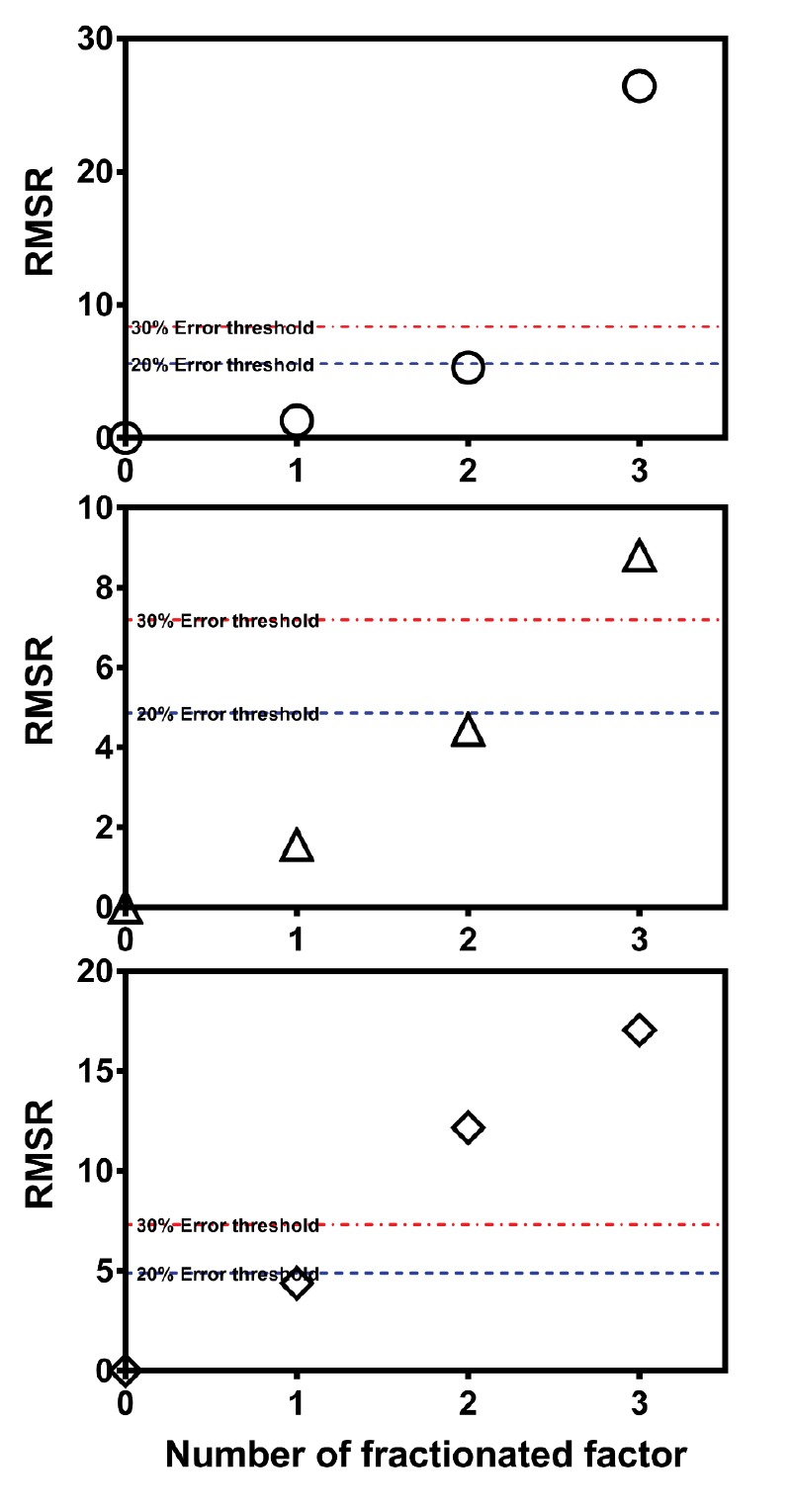
Root mean square of residual (RMSR) plot of %transmittance (O), emulsification time (△) and drug load (⋄). Number of fractionated factor of 2^6^ full factorial design (FD) = 0, 2^6-1^ fractional factorial design (FFD) = 1, 2^6-2^ FFD = 2, and 2^6-3^ FFD = 3. ----- (20% RMSR threshold limit) and ----- (30% RMSR threshold limit).

The assessment of aberration of FFD with respect to the selection of an appropriate component depending on the residual value from full design was quantitatively performed. RMSR was calculated based on Eq. 3. The lower the RMSR, the more similar the main effect was to the full design. The RMSR plot as a function of the number of fractionated factors is presented in Figure 6. All RMSR plots featured an exponential function as an increase in the number of fractionated factors. The 2^6-1^ FFD RMSR value was less than 5% of errors from the 2^6^ full FD. Moreover, with the increase in one of the fractionated factors, the 2^6-2^ FFD RMSR value rose (less than 20% error threshold). A similar pattern was observed for the ET RMSR value. However, the DL RMSR value exhibited a different pattern because certain important runs were missing from the full design. The different patterns of the RMSR plots were mainly caused by the data for each response. Statistically, missing data in reduction runs during fractional design increased the residual value and led to inadequate information.^26^ Nevertheless, compared to the solubility study followed by the ternary diagram model, the use of FFD was the best choice to select an appropriate component in the SNEDDS formulation. Despite the main effects and interaction, FFD was more efficient compared to one factor at one time, e.g., solubility in each component, followed by miscibility of oil, surfactant, and co-surfactant.

**Figure 7:**
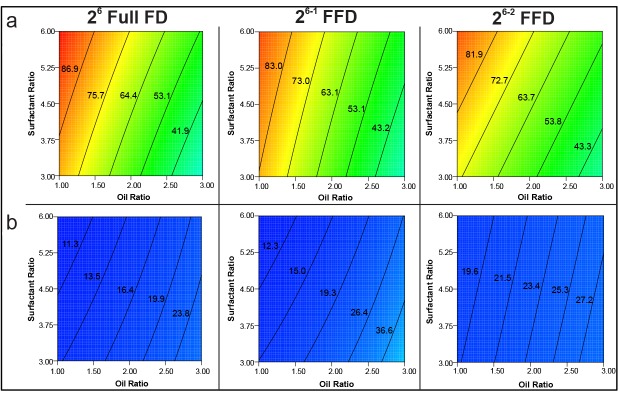
Contour plot of (a) %transmittance using Capryol, Tween 80, and Tanscutol CG at the low level and (b) emulsification time using oleic acid, Tween 80 and Transcutol P at the high level. Under comparison of 2^6^ full factorial design (FD), 2^6-1^ Fractional FD (FFD), and 2^6-2^ FFD

**Figure 8:**
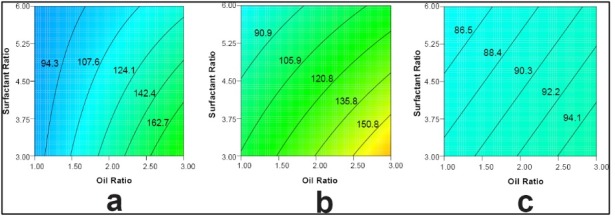
Contour plot of pitavastatin load using oleic acid, Tween 80, and Transcutol CG at the low level with different designs: (a) 2^6^ full factorial design (FD), (b)2^6-1^ Fractional FD (FFD), and (c) 2^6-2^ FFD.

To elucidate the main effect or interaction, a contour plot was constructed depending on the equation for each response.^41^ In particular, the contour plot of each design was compared with one another. Of relevance, the contour plot of %T is located in Figure 7a. It was constructed to elucidate the effect of surfactant ratio and oil ratio using capryol as oil, Tween 80 as surfactant, and transcutol P as co-surfactant at a low level. The patterns of the contour plots of both main effect pattern and contour line value were quite alike. The contour plot for ET is presented in Figure 7b. It shows the effect of oil ratio and surfactant ratio on ET using oleic acid as oil, Tween 80 as surfactant, and transcutol P as co-surfactant at a high level. All contour plots had similar patterns for the main effect. However, the contour line value in the 2^6-2^ FFD contour plot differed slightly from that in either the full design or 2^6-1^ FFD. The contour plot of DL using oleic acid, Tween 80, and transcutol P at a low level is featured in Figure 8. The contour plots of the 2^6^ full FD and 2^6-1^ FFD had a similar pattern but different contour values. Moreover, the 2^6-2^ FFD had a different contour pattern and value. These results can be explained by the missing data during reduction in runs as mentioned earlier. Based on the screening data for the selection of appropriate components in SNEDDS formulations, capryol, Tween 80, and transcutol P were chosen. However, the 2^6-2^FFD did not seem adequate enough for optimization, thus the 2^6^ FD was applied for optimization. For instance, model evaluation can be observed in actual and predicted plots of the 2^6^ full FD (Figure S4). According to this design evaluation (overlay plot, Figure S5), capryol-90 of 9.9%, Tween 80 of 50.5%, and transcutol P of 39.6% were selected for an optimized formulation, which had a particle size of 69.7 ± 5.3 nm along with a zeta potential of 33.4 ± 2.1 mV, respectively (Figure S6). In addition, a clear and bluish appearance (%T of 95%) of the nanoemulsion was evident when the optimized formulation was diluted with medium with a short ET (6.9 ± 2.5 s). Therefore, the optimized formulation was developed by 2^6^ FD according to the selected component that was screened with the 2^6-2^ FD, thereby fulfilling the requirement of self-nanoemulsion characteristics.^8,16,19^

## Conclusion

The effectiveness of FFD in screening and selection of appropriate components in SNEDD formulations has been studied extensively. Reducing the number of factors induces a lack of information exponentially. However, the 2^6-1^ FFD had a similar pattern for both contribution effects and ranking pattern. Therefore, the 2^6-1^ FFD depicted full FFD. Moreover, the 2^6-2^ FFD was the best approach for the selection of appropriate components in the SNEDDS formulation owing to its efficiency in reducing the number of runs without a lack of main effect data. Owing to a reduction in half factor number, the 2^6-3^ FFD exhibited a deficit in both contribution effect and prediction of response. Therefore, in the screening of SNEDD composition, it is our contention that the 2^6-2^ FFD could be used to select the appropriate components to develop the design space region in SNEDDS formulations.

## Ethical Issues

Not applicable.

## Conflict of Interest

All authors declare there was no conflict of interest.

## Acknowledgments

This research was funded by “Beasiswa Unggulan Dosen Indonesia (BUDI)”, Indonesian Endowment Fund for Education (LPDP). The authors would like to thank Gattefose (Saint-Priest, France) and BASF (Ludwigshafen, Germany) for providing the excipients. Syaiful Choiri was also would like to thank LPDP for his scholarship.

## Supplementary files


Supplementary file 1 contains Table S1-S2 and Figures S1-S6.
