# Supplementary material for: Assessment of Fractional Factorial Design for the Selection and Screening of Appropriate Components of a Self-nanoemulsifying Drug Delivery System Formulation
Source: Adv Pharm Bull. 2019 Oct 24;9(4):609–18. doi: 10.15171/apb.2019.070 (PMC6912180; doi:10.15171/apb.2019.070)
Supplement: Supplementary file 1 — contains Table S1-S2 and Figures S1-S6. [file apb-9-609-s001.pdf]

Supplementary File 1

Table S1. Design models using 2^6 full factorial design (FD) (64 runs), 2^{6-1} fractional FD (FFD) (32 runs and highlighted with red box), 2^{6-2} FFD (16 runs and highlighted with blue box) and 2^{6-3} FFD (8 runs and highlighted with green box)

No. Run	Categorical Factor			Numeric factor			Fractional factorial design		
	A	B	C	D	E	F	2^{6-1}	2^{6-2}	2^{6-3}
1	Capryol	Tween 80	PEG 400	1	6	4			
2	Capryol	Tween 80	PEG 400	1	3	1			
3	Oleic acid	Tween 80	Transcutol CG	1	6	1			
4	Capryol	Tween 80	Transcutol CG	3	6	1			
5	Oleic acid	Kolliphor EL	PEG 400	3	6	4			
6	Capryol	Kolliphor EL	PEG 400	3	6	4			
7	Capryol	Kolliphor EL	PEG 400	1	3	1			
8	Oleic acid	Tween 80	PEG 400	3	6	4			
9	Oleic acid	Tween 80	PEG 400	3	3	1			
10	Capryol	Kolliphor EL	Transcutol CG	1	6	1			
11	Oleic acid	Tween 80	Transcutol CG	1	6	4			
12	Capryol	Tween 80	Transcutol CG	1	3	4			
13	Capryol	Kolliphor EL	Transcutol CG	1	3	1			
14	Capryol	Tween 80	PEG 400	3	3	4			
15	Oleic acid	Kolliphor EL	Transcutol CG	3	6	4			
16	Oleic acid	Tween 80	Transcutol CG	3	6	4			
17	Oleic acid	Kolliphor EL	PEG 400	3	3	1			
18	Capryol	Tween 80	PEG 400	3	6	4			
19	Capryol	Kolliphor EL	Transcutol CG	3	3	1			
20	Oleic acid	Tween 80	Transcutol CG	1	3	1			
21	Oleic acid	Kolliphor EL	Transcutol CG	3	3	4			
22	Capryol	Kolliphor EL	PEG 400	3	6	1			
23	Capryol	Kolliphor EL	PEG 400	1	6	4			
24	Oleic acid	Tween 80	PEG 400	3	6	1			
25	Capryol	Tween 80	PEG 400	3	3	1			
26	Capryol	Tween 80	PEG 400	1	6	1			
27	Capryol	Tween 80	Transcutol CG	3	3	4			
28	Capryol	Tween 80	Transcutol CG	3	6	4			
29	Capryol	Tween 80	PEG 400	1	3	4			
30	Oleic acid	Kolliphor EL	PEG 400	1	3	1			
31	Capryol	Tween 80	Transcutol CG	1	6	4			
32	Oleic acid	Tween 80	Transcutol CG	3	3	1			

No. Run	Categorical Factor			Numeric factor			Fractional design		factorial
	A	B	C	D	E	F	2^{6-1}	2^{6-2}	2^{6-3}
33	Oleic acid	Kolliphor EL	PEG 400	1	6	4			
34	Oleic acid	Kolliphor EL	PEG 400	3	3	4			
35	Oleic acid	Tween 80	PEG 400	1	6	4			
36	Oleic acid	Tween 80	Transcutol CG	1	3	4			
37	Oleic acid	Kolliphor EL	Transcutol CG	1	3	4			
38	Capryol	Kolliphor EL	Transcutol CG	1	6	4			
39	Oleic acid	Kolliphor EL	Transcutol CG	1	3	1			
40	Oleic acid	Tween 80	PEG 400	1	3	1			
41	Oleic acid	Kolliphor EL	Transcutol CG	3	6	1			
42	Capryol	Kolliphor EL	PEG 400	1	6	1			
43	Capryol	Kolliphor EL	Transcutol CG	3	6	1			
44	Oleic acid	Kolliphor EL	PEG 400	1	3	4			
45	Capryol	Kolliphor EL	Transcutol CG	3	3	4			
46	Capryol	Tween 80	PEG 400	3	6	1			
47	Oleic acid	Tween 80	Transcutol CG	3	6	1			
48	Oleic acid	Kolliphor EL	Transcutol CG	1	6	4			
49	Oleic acid	Tween 80	PEG 400	1	3	4			
50	Capryol	Kolliphor EL	PEG 400	1	3	4			
51	Capryol	Kolliphor EL	Transcutol CG	3	6	4			
52	Oleic acid	Kolliphor EL	Transcutol CG	1	6	1			
53	Oleic acid	Tween 80	PEG 400	1	6	1			
54	Oleic acid	Tween 80	Transcutol CG	3	3	4			
55	Capryol	Tween 80	Transcutol CG	1	3	1			
56	Capryol	Kolliphor EL	Transcutol CG	1	3	4			
57	Capryol	Tween 80	Transcutol CG	3	3	1			
58	Oleic acid	Kolliphor EL	PEG 400	1	6	1			
59	Capryol	Kolliphor EL	PEG 400	3	3	4			
60	Oleic acid	Tween 80	PEG 400	3	3	4			
61	Oleic acid	Kolliphor EL	Transcutol CG	3	3	1			
62	Oleic acid	Kolliphor EL	PEG 400	3	6	1			
63	Capryol	Kolliphor EL	PEG 400	3	3	1			
64	Capryol	Tween 80	Transcutol CG	1	6	1			

A = oil types
B = surfactant types
C = co-surfactant types
D = oil ratio
E = surfactant ratio
F = co-surfactant ratio

Table S2. Observed value of %transmittance, emulsification time, and drug load

No Runs	%Transmittance (%)	Emulsification Time (s)	Drug Load (mg/mL)
1	90.30±1.21	29.29±1.81	76.78±2.51
2	39.30±4.82	93.36±13.91	39.74±1.01
3	90.47±0.81	19.19±1.58	54.13±1.87
4	47.90±2.29	25.20±1.93	44.54±4.50
5	91.37±1.90	14.19±0.35	140.02±1.76
6	42.10±0.78	199.55±12.24	107.58±2.25
7	85.10±0.20	8.33±0.14	74.00±1.69
8	47.27±1.70	67.56±2.66	60.85±0.88
9	60.77±1.44	18.23±0.72	119.20±5.40
10	1.53±0.38	18.39±2.82	183.59±9.72
11	21.57±4.75	86.37±1.05	131.57±5.97
12	8.17±1.10	179.05±42.08	175.72±5.17
13	78.73±0.40	12.49±1.97	171.59±2.18
14	1.97±0.12	73.79±7.38	113.92±6.77
15	11.60±3.15	131.48±5.36	216.21±6.07
16	8.30±1.73	127.46±17.27	160.56±2.59
17	99.10±0.20	17.68±1.31	66.61±1.36
18	54.17±0.81	223.74±12.77	85.99±1.32
19	99.57±0.23	41.60±1.63	37.34±1.00
20	61.83±2.66	87.55±4.35	45.40±2.08
21	99.03±0.21	11.29±1.32	60.85±4.48
22	65.57±0.32	51.48±2.30	51.06±2.91
23	99.60±0.36	8.94±0.63	132.53±1.16
24	55.90±0.30	36.05±5.72	80.04±11.65
25	66.67±2.93	35.34±6.59	46.36±1.16
26	27.10±2.85	36.21±5.09	79.18±2.67
27	65.60±1.71	21.33±3.69	47.90±0.44
28	11.93±0.15	21.37±2.86	99.91±5.94
29	66.20±0.85	37.22±3.12	72.75±1.04
30	33.17±8.48	106.99±3.32	50.49±0.72
31	64.00±2.77	17.98±1.22	108.45±6.25
32	13.70±2.99	28.29±1.41	134.55±1.66
33	88.77±0.96	15.03±2.32	78.22±8.12
34	43.77±1.35	59.67±7.30	38.97±1.45
35	88.17±1.86	14.74±1.57	59.22±2.08
36	49.60±0.26	46.54±4.03	55.76±0.76
37	93.03±0.67	8.53±0.73	142.23±1.45
38	39.77±0.46	21.16±4.00	69.10±2.18
39	92.73±1.14	4.65±0.78	159.88±1.74

No Runs	%Transmittance (%)	Emulsification Time (s)	Drug Load (mg/mL)
40	40.47±2.84	14.97±2.17	309.01±18.34
41	67.27±0.81	22.35±1.46	70.73±1.32
42	3.17±0.64	42.28±4.25	109.31±7.76
43	34.27±3.61	85.02±6.84	65.65±3.93
44	17.43±2.17	60.60±5.28	59.60±0.60
45	89.23±0.90	5.13±0.36	105.86±1.75
46	4.23±0.40	22.86±2.25	175.43±4.75
47	12.20±0.50	10.55±0.18	196.16±2.94
48	0.73±0.15	16.54±1.54	176.87±2.80
49	98.57±0.12	9.90±0.53	69.29±5.99
50	57.27±7.68	84.24±3.32	43.00±1.01
51	99.23±0.15	8.12±0.61	85.42±1.75
52	58.87±4.53	26.75±3.88	57.78±3.49
53	99.43±0.21	6.52±0.88	88.39±3.27
54	68.40±0.70	8.70±0.42	51.45±0.58
55	99.03±0.25	5.19±0.68	177.35±4.57
56	65.20±2.31	8.51±1.16	240.68±8.33
57	71.97±2.85	47.87±3.56	53.75±0.76
58	34.40±0.70	29.78±4.34	58.26±0.60
59	57.27±4.05	13.11±0.43	82.63±0.83
60	32.43±3.36	56.62±7.58	47.42±0.76
61	63.33±1.12	10.39±1.30	118.91±3.17
62	27.77±3.96	41.65±5.96	119.87±1.87
63	68.73±1.89	15.11±1.61	132.92±2.75
64	24.33±0.50	36.04±4.56	171.59±5.72

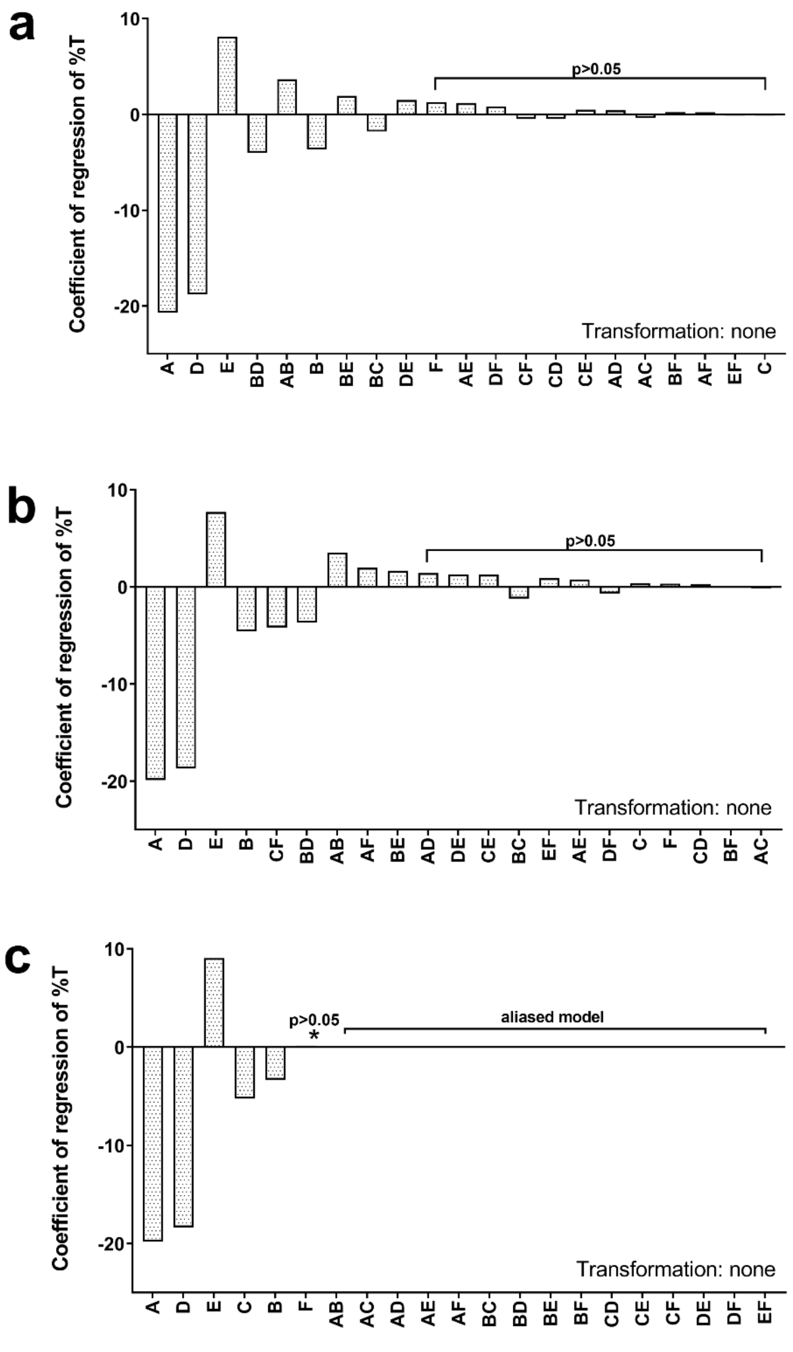

Figure S1. Pareto chart of main effect and interaction of transmittance using 2^6 full factorial design (FD) (a), 2^{6-1} fractional FD (FFD) (b), and 2^{6-2} FFD (c)
Non-significant main effect and interaction assigned with p>0.05. Interaction model was disable owing to aliased term in modelling parameters.

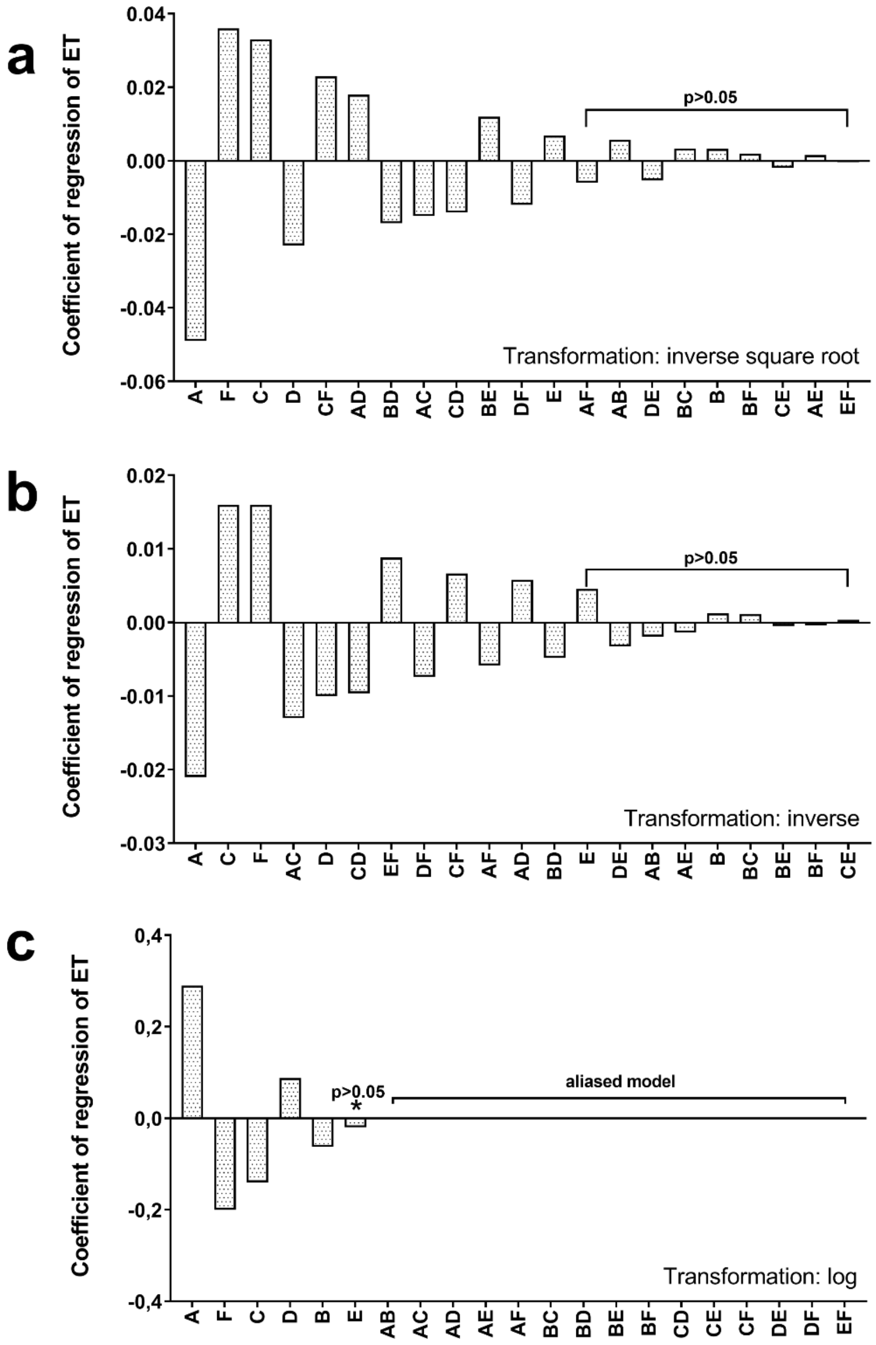

Figure S2. Pareto chart of main effect and interaction of emulsification time (ET) using 2^6 full factorial design (FD) (a), 2^{6-1} fractional FD (FFD) (b), and 2^{6-2} FFD (c) Non-significant main effect and interaction assigned with p>0.05. Interaction model was disable owing to aliased term in modelling parameters.

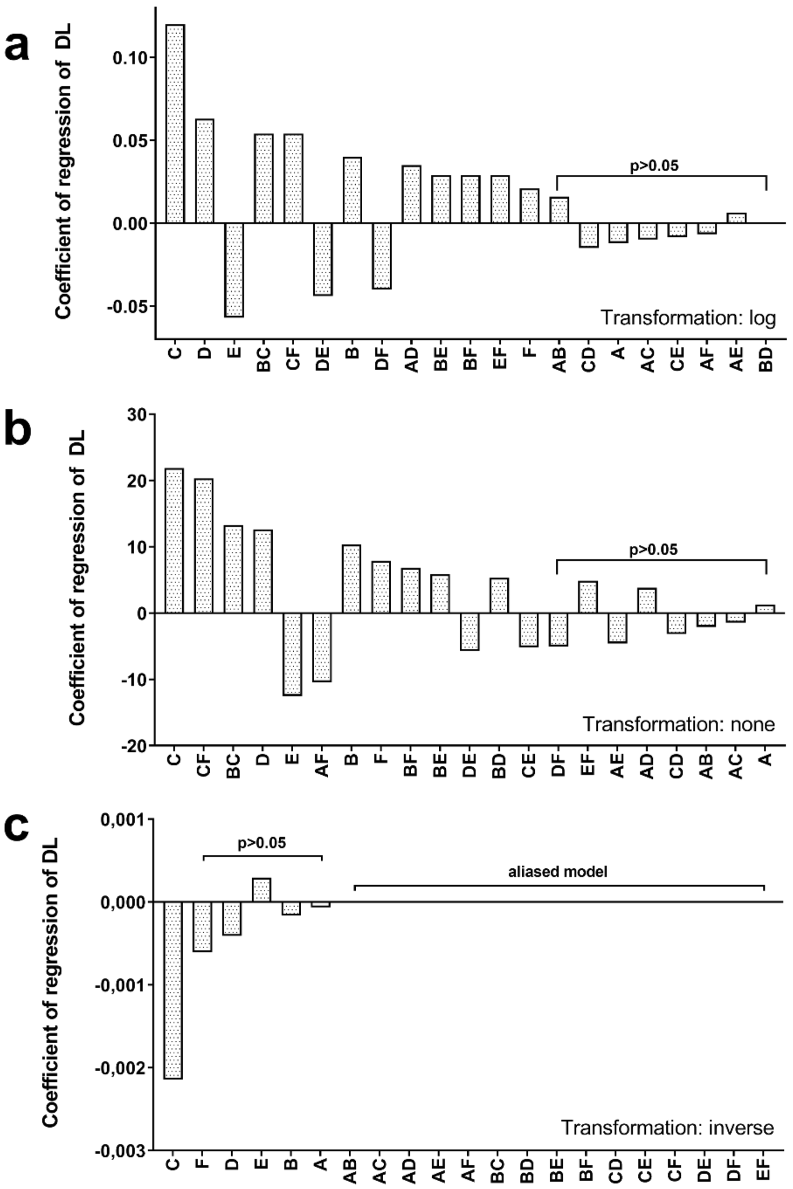

Figure S3. Pareto chart of main effect and interaction of drug loading (DL) using 2^6 full factorial design (FD) (a), 2^{6-1} fractional FD (FFD) (b), and 2^{6-2} FFD (c)
Non-significant main effect and interaction assigned with p>0.05. Interaction model was disable owing to aliased term in modelling parameters.

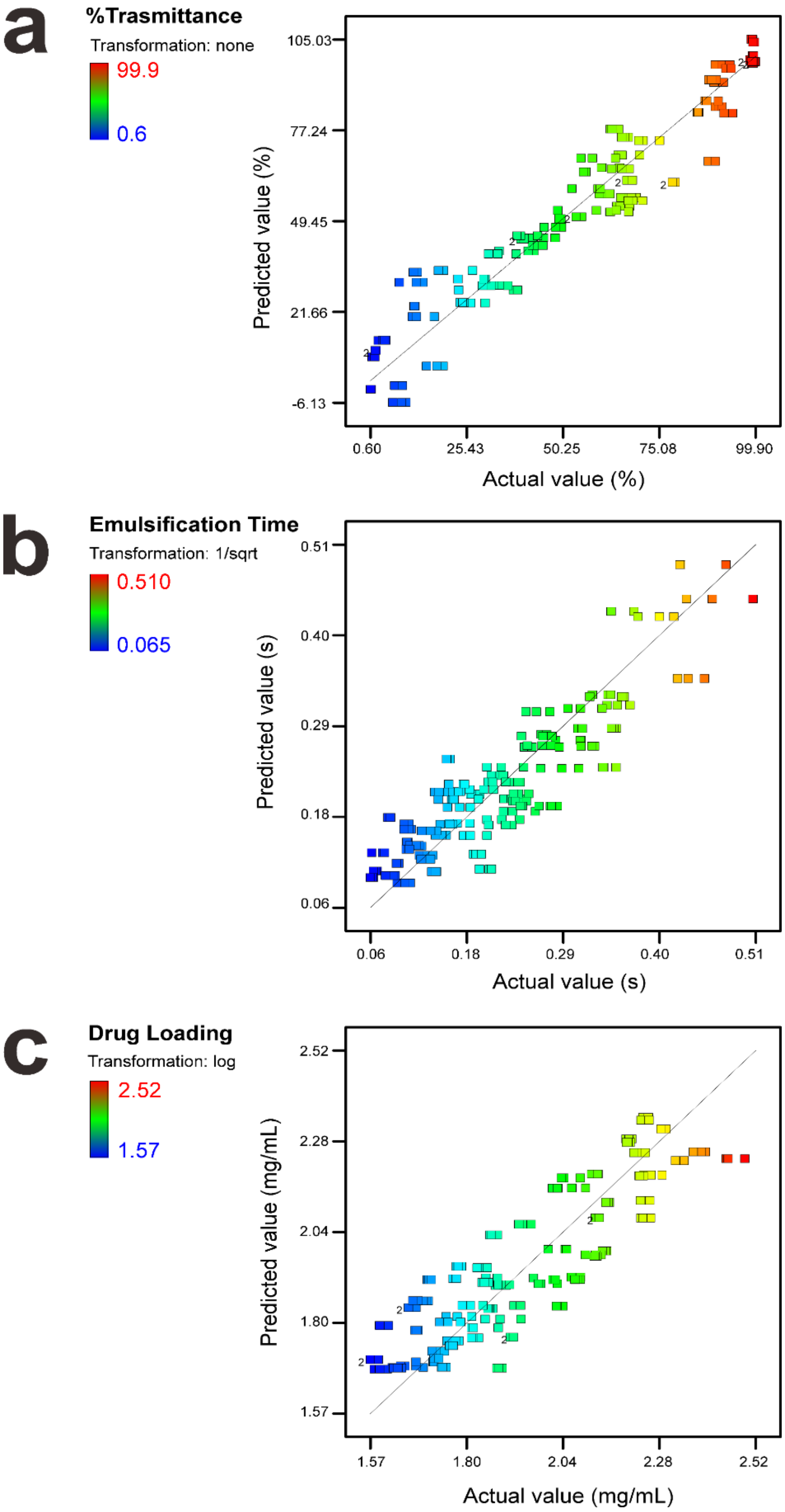

Figure S4. Actual vs predicted plot of 2^6 full factorial design. (a) %transmittance, (b) emulsification time, and (c) drug load

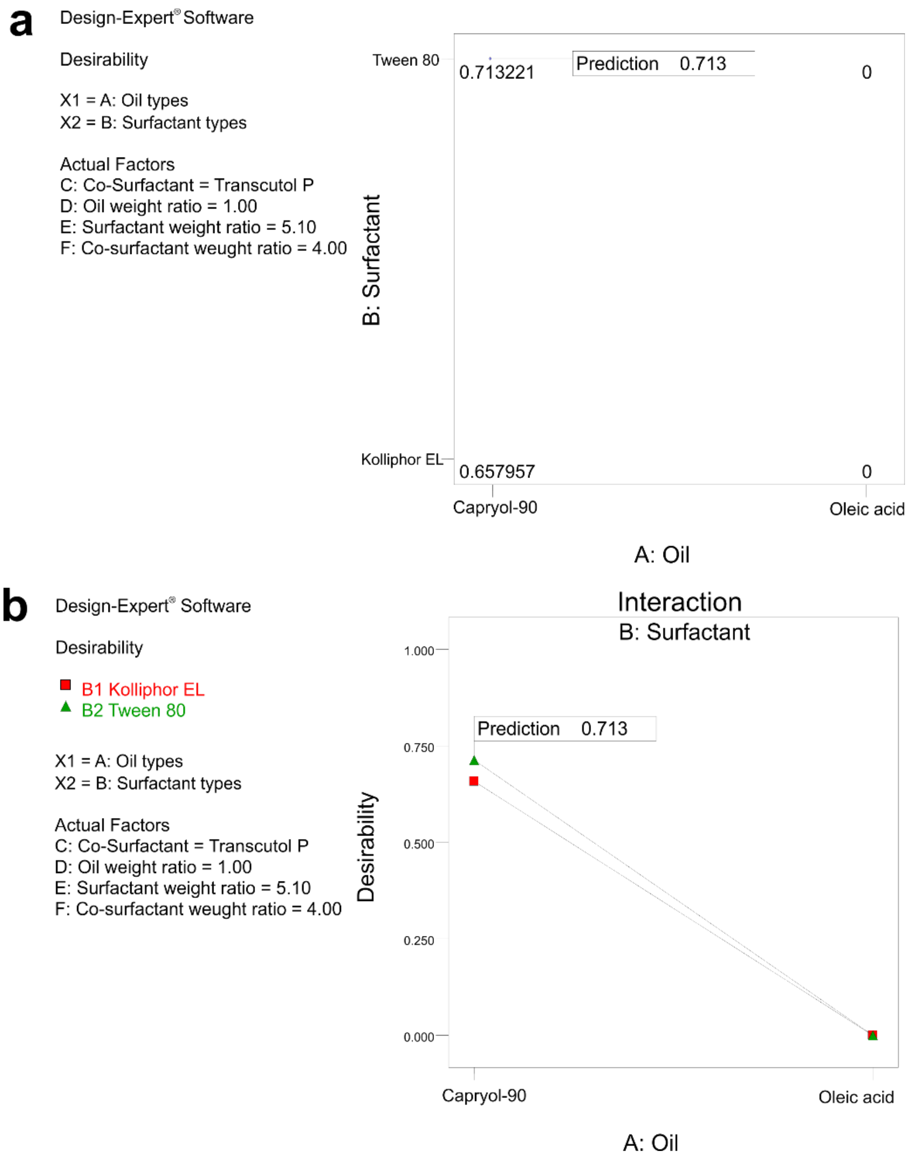

Figure S5. Optimized formulation determined by over-lay plot (a) and interaction plot (b).

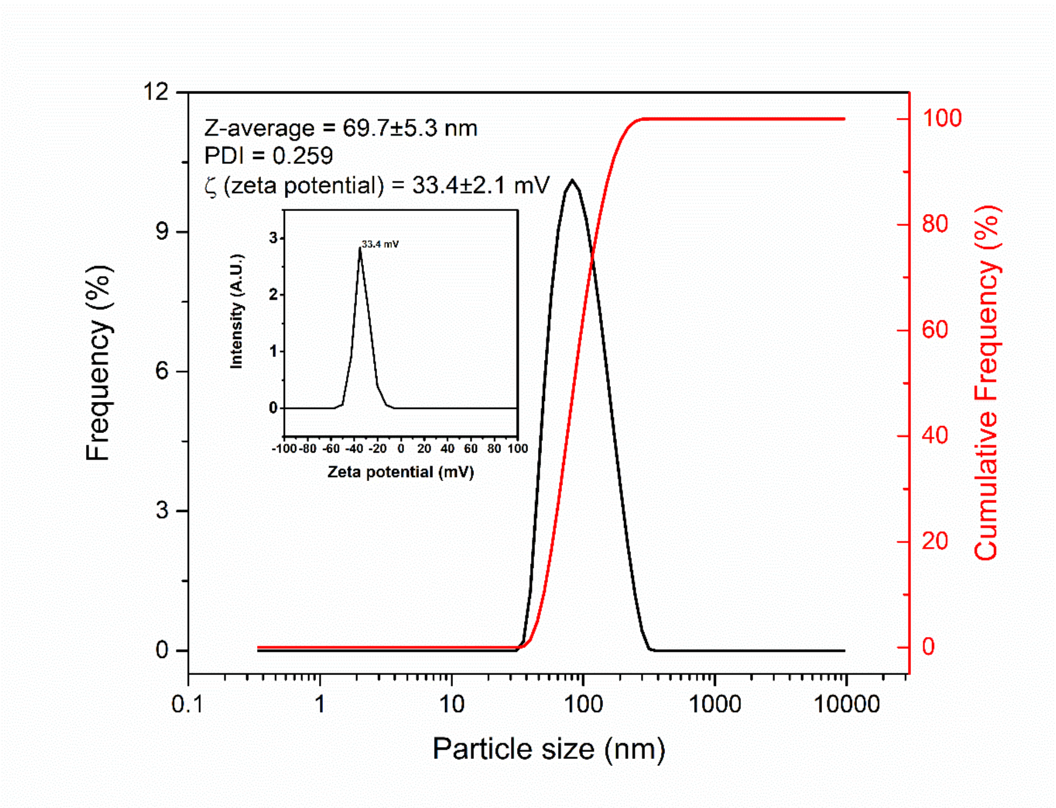

Figure S6. Particle size along with zeta potential characterization for optimized formulation.
